# Enhancing the delivery of comprehensive care for people living with HIV in Canada: insights from citizen panels and a national stakeholder dialogue

**DOI:** 10.1186/s12961-024-01147-1

**Published:** 2024-05-27

**Authors:** Michael G. Wilson, Cristina Mattison, Kerry Waddell, Jean Bacon, Marissa Becker, Christine Bibeau, John N. Lavis, Ron Rosenes, Claire E. Kendall

**Affiliations:** 1 McMaster Health Forum, McMaster University, 1280 Main Street West, MML-417, Hamilton, ON L8S 4L6 Canada; 2 Department of Health Research Methods, Evidence and Impact, McMaster University, Hamilton, Canada; 3 Centre for Health Economics and Policy Analysis, McMaster University, Hamilton, Canada; 4 Women’s and Children’s Health, Karolinska Institut, Stockholm, Sweden; 5 Ontario HIV Treatment Network, Toronto, Canada; 6 Departments of Medicine, Medical Microbiology, and Community Health Sciences, University of Manitoba, Winnipeg, Canada; 7 C.T. Lamont Primary Health Care Research Centre, Bruyère Research Institute, Ottawa, Canada; 8 Department of Political Science, McMaster University, Hamilton, Canada; 9 Department of Family Medicine, University of Ottawa, Ottawa, Canada

**Keywords:** HIV, Primary care, Comprehensive care, Citizen engagement, Stakeholder dialogue

## Abstract

**Background:**

People living with human immunodeficiency virus (HIV) are living longer with health-related disability associated with ageing, including complex conditions. However, health systems in Canada have not adapted to meet these comprehensive care needs.

**Methods:**

We convened three citizen panels and a national stakeholder dialogue. The panels were informed by a plain-language citizen brief that outlined data and evidence about the challenge/problem, elements of an approach for addressing it and implementation considerations. The national dialogue was informed by a more detailed version of the same brief that included a thematic analysis of the findings from the panels.

**Results:**

The 31 citizen panel participants emphasized the need for more prevention, testing and social supports, increased public education to address stigma and access to more timely data to inform system changes. The 21 system leaders emphasized the need to enhance person-centred care and for implementing learning and improvement across provinces, territories and Indigenous communities. Citizens and system leaders highlighted that policy actions need to acknowledge that HIV remains unique among conditions faced by Canadians.

**Conclusions:**

Action will require a national learning collaborative to support spread and scale of successful prevention, care and support initiatives. Such a collaborative should be grounded in a rapid-learning and improvement approach that is anchored on the needs, perspectives and aspirations of people living with HIV; driven by timely data and evidence; supported by appropriate decision supports and aligned governance, financial and delivery arrangements; and enabled with a culture of and competencies for rapid learning and improvement.

## Introduction

Many provinces and territories in Canada have prioritized health-system reforms including integrated care for people living with complex conditions across health-system sectors and with social systems [1–4]. However, care for people living with human immunodeficiency virus (HIV) poses unique challenges, such as addressing stigma and discrimination, poverty, food security, homelessness, cognitive impairments and mental health and addictions that require tailored responses [5–7]. In addition, people living with HIV who take antiretroviral medications are living longer with HIV and with more chronic conditions [8–12].

Canadian federal and some provincial governments have supported achieving (and surpassing) the 90–90–90 targets set by the Joint United Nations Programme on HIV/AIDS (UNAIDS) [13]. As of 2016, 86% of Canadians living with HIV were diagnosed; 81% of Canadians diagnosed with HIV were on treatment; and 91% of Canadians living with HIV on treatment had achieved viral suppression [13–16]. However, HIV incidence rates have not declined. Planned efforts to address these targets and incorporate prevention have been included in the Pan-Canadian Sexually Transmitted and Blood-Borne Infections (STBBI) Framework for Action, which is focussed on four pillars: (1) prevention, (2) testing, (3) linkage to care and treatment and (4) ongoing care and support [17]. To be successful, these efforts will need to focus on addressing the many complex and inter-related challenges that face people living with HIV [5, 6]. For example, as compared with other chronic diseases, HIV-related stigma is a unique consideration and has been identified as one of the biggest challenges facing people living with HIV [7]. This stigmatization has been found to be associated with increased rates of depression, lower social support, anxiety, quality of life, physical health, emotional and mental distress and trauma and sexual risk, and significantly impacts people’s ability to engage with a fragmented health system [7, 18].

## Methods

Our goal was to spark action to address these challenges by convening deliberations across Canada with people living with, at risk of and affected by HIV, and with system leaders (policymakers, stakeholders and researchers) who could champion needed changes. To do this, we convened: (1) citizen panels with people living with, at risk of and affected by HIV to learn about their views of and experiences in relation to these challenges and their values and preferences for policy options to address the challenges and (2) a national stakeholder dialogue with HIV health system leaders to identify steps that can be taken to address the challenges. The approaches used for each are described in detail in the separate documents we have published, which include a citizen brief, panels summary, evidence brief and dialogue summary [19–22]. We provide a concise overview of our approach below. In this paper, we integrate the findings from both components to provide insights from citizens and system leaders about the important next steps identified for enhancing comprehensive care for people living with HIV in Canada.

The project was led by an interdisciplinary steering committee, which included people living with HIV as well as policymakers, clinicians and researchers with expertise in HIV-related policy, care and support. The committee guided the project from start to finish, including establishing the scope of the project, providing feedback on the citizen and evidence brief, identifying key informants to engage to provide feedback on the citizen and evidence brief, shaping the recruitment criteria for the citizen panels, identifying participants for the stakeholder dialogue and providing feedback on the analysis of the deliberations.

First, we convened three citizen panels in 2019 in Manitoba (March 22), Ontario (April 5) and Newfoundland and Labrador (April 22) that sought to engage participants from each Canadian province. Each panel was informed by a plain-language citizen brief that described what is known on the basis of data and evidence and insights from 25 key informants [19, 21]. The deliberations focussed on describing the underlying problem related to enhancing comprehensive care for people living with HIV in Canada, three possible elements of an approach to addressing the problem, and implementation considerations for the elements. Panellists were recruited through the AskingCanadians™ panels, which include more than 600 000 Canadians that are affiliated with loyalty programs in Canada and are representative of all the Statistics Canada demographic categories. We sought to engage 14–16 panellists living with or affected by HIV for each panel who were diverse in terms of gender, age, sexual orientation, socioeconomic status, ethnocultural background and geographic residence (both in terms of provinces they live in and from urban, suburban and rural/remote settings). The deliberations were facilitated by one of two team members (MGW and CM) and followed the structure of the citizen brief. We summarized key insights from the panel using themes that the facilitators identified and refined from their detailed notes in each panel. Within panels, these themes were discussed and refined with panellists through mid-day summaries that were reviewed and discussed. In addition, our wrap-up deliberations for each were used to debrief with the panellists to ensure that we accurately documented and understood the experiences, values and preferences that were articulated during the deliberations. We also used this time to ensure that areas of common ground and divergence were accurately captured and framed in a way that reflected the deliberations. We continued to identify and refine themes through subsequent panels and used our detailed notes to capture areas of consistency across panels, as well as insights that were unique to a particular panel. After all of the panels were convened, we selectively revisited recordings to ensure accuracy of notes, themes identified and illustrative quotes.

In May 2019, we convened a stakeholder dialogue that was informed by pre-circulated evidence brief [22] (a more detailed version of the citizen brief), which included key findings from the panels. We identified participants in collaboration with the project steering committee on the basis of their ability to: (1) bring unique views, experiences and tacit knowledge to bear on the challenge and learn from the research evidence and from others’ views, experiences and tacit knowledge and (2) champion actions that will address the challenge creatively. The dialogue concluded with a focus on potential next steps that could be taken. Deliberations were facilitated by one of us (MGW) with detailed notes and observations taken by the facilitator and one secretariat (KW), which were used to prepare an analysis of key insights from the deliberations [20]. This included a draft summary of key themes that we provided to all dialogue participants to reflect on before the dialogue was completed. The stakeholder dialogue was convened “off the record” and adhered to the Chatham House Rule (participants are free to use the information received, but neither the identity nor the affiliation of the speaker(s), nor that of any other participant, may be revealed) and therefore deliberations were not recorded.

### Key insights from citizen and stakeholder engagement

The citizen panels convened 31 ethnoculturally and socio-economically diverse people living with or affected by HIV from all provinces except Saskatchewan (demographic information provided in the panel summary) [19]. Demographic information of panellists is provided in Table 1. The stakeholder dialogue convened 21 participants, which included people living with HIV and Indigenous people. While most participants held several positions, the principal roles included five federal- and provincial-level policymakers and/or leaders of a health region, five leaders of community-based HIV organizations, five leaders of stakeholder groups (including professional organizations, national and provincial-level groups and citizen-based groups) and six researchers (most of whom were also clinicians).

**Table 1:** Profile of citizen panel participants (*n* = 31)

Categories	*N*
Province	
British Columbia	1
Alberta	5
Saskatchewan	0
Manitoba	7
Ontario	10
Quebec	4
New Brunswick	2
Nova Scotia	1
Prince Edward Island	1
Newfoundland and Labrador	0
Region	
Urban	15
Suburban	8
Rural	8
Age, years	
25–44	8
45–64	15
65 and older	8
Self-identified gender	
Women	21
Men	10
Not identified as male or female	0
Self-identified ethnic background	
Canadian	17
European	5
Indigenous	4
Asian	1
West Indian/Caribbean	1
Other (specified as Arab, Latino, Métis)	3
Education	
Bachelor’s degree	9
Post-graduate training or professional degree	6
Community college	6
High school	6
Technical school	4
Work status	
Working full time	10
Working part time	5
Self-employed	2
Unemployed	2
Retired	8
Disabled	4
Income	
< $20 000	4
$20 000–40 000	8
$40 000–60 000	3
$60 000–80 000	3
> $80 000	8
Preferred not to answer	5

#### Insights about challenges related to the problem

Panellists identified eight challenges, which are summarized in Table 2 and include: (1) lack of comprehensive supports for HIV prevention, (2) limited access to point-of-care testing, (3) stigma is pervasive and layered, (4) lack of public awareness and education to address stigma, (5) privacy and confidentiality in testing and care are not respected, (6) limited access to social-systems supports, (7) the problem is magnified for the most vulnerable and (8) lack of timely data and use of evidence in policy decisions.

**Table 2:** Summary of citizens’ views about challenges

Challenge	Description
Lack of comprehensive supports for HIV prevention	• Panellists raised three challenges related to comprehensive HIV prevention: ○ limited investments in inexpensive but highly effective forms of prevention (e.g. harm reduction); ○ lack of access, coverage for and health professional knowledge about pre-exposure prophylaxis (e.g. Truvada); and ○ existing models present barriers to effective testing and prevention (e.g. limited number of anonymous testing services) • Panellists emphasized that these prevention challenges are magnified for marginalized and stigmatized populations (e.g. Indigenous peoples, and particularly those living in remote communities; people who inject drugs; and people who are incarcerated)
Limited access to point-of-care testing	• Many panellists expressed frustration with challenges in accessing point-of-care testing, but there was variability in the concerns raised by panellists across the citizen panels • Several panellists also questioned why access to home-based self-testing cannot be made available, while others expressed concern with this approach to testing given the lack of direct linkage to needed care and supports following a positive diagnosis • In the Winnipeg citizen panel, panellists noted that while there are sexually transmitted infection clinics, many have long wait lists and are only open during business hours, which creates barriers to timely access to point-of-care testing • In the Hamilton citizen panel, panellists shared some positive experiences with accessing point-of-care testing (e.g. in settings such as the Hassle Free Clinic), but they had concerns regarding the anonymity of the process ○ Specifically, while accessing point-of-care testing is anonymous, panellists were concerned that positive HIV test results are reported to the local public-health authorities and about their perceived lack of control over whether and how their health information is shared • In the St. John’s citizen panel, panellists were most concerned with the overall lack of point-of-care testing in Atlantic provinces
Stigma is pervasive and layered	• Most of the panellists felt that stigma is pervasive and can lead to overt forms of discrimination • Panellists indicated that stigma is a key reason why HIV is different than other chronic conditions ○ One participant in the Hamilton panel shared that other chronic conditions would not have led them to not be able to live in their home in a rural community where the fear of being stigmatized and discriminated against is significant • Stigma was described as layered and that individuals may live with multiple forms of stigma (e.g. people living with HIV who are gay), which can create significant barriers to care, including testing and engaging in care • A few panellists experienced stigma by health professionals after requesting HIV testing and thought that this type of stigma can also lead to avoiding testing • The criminalization of HIV non-disclosure was raised by a number of participants as contributing to the increased stigmatization of living with HIV • Panellists felt that Indigenous peoples were the most marginalized and stigmatized of all the groups discussed • One panellist described challenges with social inclusion and provided an example of experiencing stigma when trying to find faith-based support in the community after diagnosis
Lack of public awareness and education to address stigma	• Many panellists described an overall lack of “social education” as perpetuating stigma, which is closely linked with the previous challenge • High school health education was felt to be fear-based and perpetuated the stigma associated with sexually transmitted infections • Panellists also thought that sex education was happening too late in high school and that education was needed in middle school • A few panellists also had concerns that HIV is no longer viewed by the public as a problem and that the awareness generated in the 1980s and 1990s has been lost • Similarly, some panellists felt that pre-exposure prophylaxis may give a false sense of security and that education in this area was lacking
Privacy and confidentiality in testing and care are not respected	• A number of panellists had concerns with privacy and confidentiality related to seeking HIV testing or care in rural and remote communities • Some did not trust that their results would remain confidential and feared that health professionals or administrators within primary-care practices would disclose HIV status to the patient’s family or members of the community • One panellist cited this as the reason for leaving the small community and seeking care in a large city
Limited access to social-system supports	• Panellists expressed that limited access to social-system supports was one of the biggest barriers to enhancing comprehensive care for people living with HIV • Social-system supports were described as a core component, above health considerations, and one panellist summarized the point as “you need the basics, it’s survival.” • Panellists described limitations with community capacity, primarily lack of opportunities for meaningful engagement in policy/governance as well as ability for self-determination to derive culturally appropriate policy and programs across health and social systems
Problem is magnified for the most vulnerable	• All of the challenges are magnified for the most vulnerable, including those whose basic needs are not being met, Indigenous peoples, people who are or have been incarcerated and/or people who use drugs • Social and structural challenges faced by vulnerable populations make it hard to be tested and/or engaged and retained in care
Lack of timely data and use of evidence in policy decisions	• A few panellists were frustrated with the lack of timely data in Canada and lack of consistency and standards in data collection across provinces and territories • The lack of timely Canadian data was also found to hinder cross-country comparisons regarding the 90–90–90 targets (e.g. the UNAIDS country fact sheet for Canada is empty) • Panellists also thought that research evidence was not used in many policy decisions and gave the example of point-of-care testing, noting that if decisions were based on evidence then the testing would be available more broadly

Dialogue participants agreed with these challenges, and expanded the list to include:

1. social and structural challenges including stigma and discrimination are fundamental to address, but continue to lack traction and commitment to change;
2. lack of emphasis on prevention and making available a broad menu of testing options that meet the needs of different communities;
3. lack of coordination across care pathways and throughout the lifespan;
4. limited access to timely data and the many different forms of evidence needed to inform policy and programmatic decision-making; and
5. mechanisms and resources not being in place to support learning across provinces, territories and Indigenous communities.

#### Insights about elements of a potentially comprehensive approach to address the problem

Citizen panellists and dialogue participants deliberated about three pre-circulated elements of a potentially comprehensive approach to address the problem: (1) strengthening comprehensive HIV care within the health system; (2) providing supports across social systems to address all of the challenges faced by people living with HIV; and (3) adopting a rapid-learning and improvement approach to incrementally strengthen health and social systems. These elements and evidence about them are detailed in the publicly available evidence brief [22]. Values and preferences from citizens and insights from system leaders are summarized in Table 3.

**Table 3:** Insights from citizens and dialogue participants about three elements of a potential approach to enhancing the delivery of comprehensive care for people living with HIV in Canada

Elements	Citizens’ values and preferences	Insights from dialogue participants
Strengthening comprehensive HIV care within the health system	• Our analysis identified five values that citizens prioritized in relation to this element, which emphasized: ○ fairness/equity in access to health services ○ empowerment (e.g. for self-advocacy); ○ privacy (e.g. for HIV test results); ○ trusting relationships between patients, health professionals and organizations within the health system; ○ collaboration among patients, health professionals and organizations within the health system; • Preferences for operationalizing these values focussed on: ○ enhancing access to comprehensive care through interprofessional team-based care and improving access to nursing stations as a site of service delivery for point-of-care testing, follow-up and counselling on treatment options; ○ ensuring privacy of testing and increasing the availability and equal access to point-of-care testing across Canada; ○ providing access to self-testing options (although views on this were mixed with some having concerns about lack of linkage to needed care supports following a positive diagnosis); ○ reducing stigma to build trusting relationships between patients and health professionals (e.g. through renewed public education efforts); ○ empowering self-advocacy through education; ○ improving electronic health records to allow for seamless transitions in care (e.g. a universally accessible electronic health record system that is easy-to-use, secure and that all parties involved in care can see); and ○ bringing care to the individual, especially for marginalized and hard-to-reach populations through mobile units or virtual care	• Participants broadly agreed that strengthening primary care should be the starting point for action • It was emphasized that primary care needs to be adaptable to meet the unique needs of people living with HIV • As a complement to the activities outlined in the evidence brief, dialogue participants also underscored the need to identify those with shared health and social challenges and developing approaches to wrap-around care that meets those needs, implement team-based approaches that bridge health and social systems, and expanding the range of providers delivering care for those with HIV
Providing supports across social systems to address all of the challenges faced by people living with HIV	• Our analysis identified three values that citizens prioritized in relation to this element, which emphasized: ○ fairness/equity in access to social services ○ trusting relationships between clients, providers and organizations within social systems; and ○ collaboration among clients, providers and organizations within social systems • Preferences for operationalizing these values focussed on: ○ combining health and social systems supports under one roof to enhance coordinated care ▪ for example, by developing and implementing community health teams for coordinating needed supports, especially following diagnosis when people are often vulnerable and need support ▪ it was emphasized that when people leave a physician’s office, they need to be connected with someone from the social system to ensure they can help with getting access to needed medications, healthy food and stable housing, as well as getting answers to questions or access to resources that are needed ○ supporting system(s) navigation through community workers or peers with lived experience (e.g. a buddy system approach was identified as being important in each of the panels, especially for smaller areas where there may not be trained people to help) ○ increasing access to affordable supportive housing as well as investments in food banks ○ combining and mobilizing existing supports (e.g. offering food when running a health clinic or needle exchange vans offering point-of-care testing)	• This element was identified as needing to be merged with element 2 for a more integrated and comprehensive approach that prioritizes providing wrap-around care from health and social systems • To do this, dialogue participants emphasized the need to allow social services to “take the lead” where appropriate, embed cultural competence, safety and responsiveness across the care continuum and support community-based organizations and community leaders to thrive to equip them to provide such leadership
Adopting a rapid-learning and improvement approach to incrementally strengthen health and social systems	• Our analysis identified three values that citizens prioritized in relation to this element, which emphasized: ○ accountability ○ collaboration among patients/clients, providers and organizations within health and social systems ○ basing decisions on data and evidence ○ basing decisions on citizens’ values and preferences ○ continuously improving (e.g. the quality of HIV-related data) ○ ensuring excellent health outcomes • Preferences for operationalizing these values focussed on: ○ need for an accountable organization(s) that can identify what changes could be made and then independently monitor and evaluate, and intervene right away to make needed changes ○ developing and implementing an interconnected database that is standardized across provinces and territories to provide timely access to continuously updated and anonymous data and evidence to promote more learning and sharing across the country ○ emphasizing local solutions that can then be adapted for use elsewhere on the basis of data and evidence and the values and preferences of citizens ○ structures to ensure processes are led by communities and meaningful engagement of people living with and affected by HIV (e.g. community councils that support people getting involved) ○ empowering communities to set their own priorities and create tailored responses to local issues ○ developing a common language to facilitate collaboration among patients, health professionals and organizations within health and social systems	• A rapid-learning orientation was viewed among all dialogue participants to be advantageous • It was viewed that moving forward with such a model would require initiatives to: ○ identify and develop an inventory of assets in health systems across the country; ○ empower individuals with advocacy skills and support their engagement in decision-making; ○ determine common datasets that could be used and shared across provinces; and ○ foster stronger lines of accountability for HIV outcomes

Overall, citizens emphasized the need to provide equitable access to integrated comprehensive care to enable people to achieve optimal outcomes regardless of where they live and the challenges they face. Moreover, citizens consistently identified supports across social systems to address the full range of challenges faced by people living with HIV as being the most fundamental, yet potentially most difficult to achieve. In addition, it was identified that actions towards strengthening social systems should be prioritized over others as it will help address challenges (e.g. housing, poverty and stigma) that put people at risk for HIV and make getting diagnosed and engaged in care challenging. Lastly, despite initially struggling with the concept of rapid-learning health systems, there was consensus about the importance of making small yet rapid changes to improve HIV care and supports over time as it was viewed as more achievable than trying to reinvent entire health and social systems.

System leaders in the stakeholder dialogue participants emphasized the need for a combined health and social-system approach to strengthening care for those with HIV. Participants specifically called for a greater role for team-based care that prioritizes mental health and addictions services, and for care that is culturally competent, safe and responsive.

They also identified four themes requiring action: (1) acknowledging that HIV continues to be unique and needs to be accounted for in the pursuit of any next steps; (2) ensuring person-centred and adaptive approaches for strengthening comprehensive HIV care in health systems and providing supports across social systems; (3) underpinning all actions taken with enhanced efforts to address stigma and normalize HIV prevention, testing, care and support; and (4) focussing on achieving the Triple Aim of excellent patient experience, improved patient outcomes and keeping per capita costs manageable.

#### Implementation considerations

Dialogue participants also identified several implementation considerations that are likely to affect efforts to champion the necessary changes. These potential barriers included: (1) funds traditionally being siloed across government programs in health and social systems, which limits the ability flow funds to support integrated whole-person care; (2) power imbalances between different organizations and stakeholders (e.g. hospitals and credentialed professionals as compared with community and non-credentialed providers), which may make integration difficult; (3) challenges in navigating different priorities and languages used across health and social systems and the sectors within them; and (4) potential perceived delays and/or lack of action due to embedding HIV targets under the broader Pan-Canadian STBBI framework.

#### Next steps identified by system leaders

Stakeholders were willing to invest in several next steps, including to:

1. focus on improving delivery of prevention, care and support and to achieve the 90–90–90 targets, adding a fourth 90 focussed on improving quality of life, and the goals in STBBI framework (while recognizing the unique and often more complex needs of those in the remaining 10% of each of the targets);
2. continue to strengthen primary-care-based models emphasizing person-centred care (and include the full complement of primary-care-based providers, such as physicians, nurses and pharmacists);
3. identify groups of individuals with shared challenges that can be collectively supported, and shared strengths that can be built upon; and
4. create wrap-around social services and primary care, and inject primary-care elements into social-systems settings (e.g. housing, supervised-injection sites and prisons).

## Discussion

Our findings highlight that policy actions across provincial and territorial health systems need to acknowledge that HIV remains unique among conditions faced by Canadians, adopt person-centred approaches to care and address HIV-related stigma. This will require grappling with many long-standing challenges for provincial and territorial health-system reforms in Canada, particularly the lack of integration between health and social care which is typically driven by funding silos, power imbalances and limited coordination which makes intersectoral care difficult to achieve. However, the STBBI framework could be used as a common galvanizing mechanism for provinces and territories for addressing these challenges. Moreover, the framework could provide common ground for establishing greater collaboration across provincial and territorial governments, such as through a learning collaborative that can support an HIV rapid-learning model.

Such a collaborative should be grounded in a rapid-learning and improvement approach [23, 24] that is anchored on the needs, perspectives and aspirations of people living with HIV; driven by timely evidence support; supported by appropriate decision supports and aligned governance, financial and delivery arrangements; and enabled with a culture of and competencies for rapid learning and improvement. This work could be grounded in the recommendations from the Global Commission on Evidence to Address Societal Challenges (GCESC), which is focussed on supporting actions to: (1) formalize and strengthen domestic evidence-support systems; (2) enhance and leverage the global evidence architecture; and (3) engage citizens, citizen leaders and citizen-serving non-governmental organizations (NGOs) in putting evidence at the centre of everyday life. In the recent update to the GCESC, rapid learning and improvement cycles were described as involving three stages [24]. The first stage focusses on making sense of a challenge or opportunity and the population(s) and then prioritizing what needs to be done. This requires continual efforts to identify where there are system gaps and what is driving them, where there are inequities and what challenges are prioritized to address. Next, the focus turns to co-designing new services and service models by identifying evidence-informed solutions that already exist, and how solutions can be adapted/designed with input from system users and communities (in this case, ensuring meaningful involvement of people living with HIV). The last stage of the cycle focusses on implementing, adapting and using system-level monitoring and evaluation (i.e. to determine what works, for whom and what adaptation are needed to support spread and scale). An HIV-focussed learning collaborative was viewed as being able to champion the type of work needed to operationalize this type of rapid-learning and improvement cycle.

Such an approach could be grounded as part of efforts to strengthen evidence-supports systems more broadly [24]. Such systems need to include: (1) structures and processes for those who can use evidence to inform decisions (i.e. on the “evidence demand” side, which can involve incorporating evidence use into routine advisory and decision-making processes, building and sustaining an evidence culture and strengthening capacity for evidence use); (2) mechanisms to enable coordination at the interface between evidence demand and the suppliers of evidence (e.g. by identifying evidence needs of decision-makers, packaging evidence from multiple sources in a way that is useful decision-making processes); and (3) evidence-support units on the evidence-supply side that have the expertise to understand the domestic context, evidence standards and preferred communication formats of decision-makers, and that are structured in a way that make them timely and demand-driven in their supply of evidence (e.g. by contextualizing existing domestic and global evidence in a way that ensures equity considerations are identified and considered) [24].

In considering these broader structures for using rapid-learning and improvement cycles and for strengthening evidence-support systems that have emerged since the deliberations were convened, there were many innovative and promising initiatives with potential for scale-up and spread in other communities, provinces and territories that were discussed during the deliberations which continue to be relevant. For example, many viewed the Ontario Health Teams as an opportunity to advance the implementation of person-centred approaches to care for people living with HIV. Similarly, a rapid-learning model is gaining traction in other provinces such as British Columbia through Michael Smith Health Research BC. Primary-care reform and addressing health human resource challenges continues to be a focal point for health-system reforms across the country and the actions described in this study can inform and be embedded within those efforts. The coronavirus disease 2019 (COVID-19) pandemic was also a catalyst for doing many things different and re-shaping priorities. In relation to this study, the focus on rapidly adjusting to address the needs of those most affected by COVID-19 can be taken as an opportunity to strengthen systems for the most marginalized and in ways that harness the ability to adjust rapidly, evaluate and continue to adjust.

## Limitations

A potential limitation of our approach is that we engaged a smaller number of citizens in the panels. We made this choice to prioritize in-depth deliberations with a purposively selected sample of citizens, which in our experience has yielded rich insights. Moreover, we were unable to engage participants from every province. Given that key themes across the three panels were consistent, we are confident that our sample size was appropriate and representative, but may not have adequately represented the needs of black, Indigenous and people of colour. In addition, these findings were used to anchor the deliberations with health and social-system leaders in the stakeholder dialogue. Doing so ensured that our approach was grounded in the experiences, values and preferences of those living with HIV and that the deliberations with health- and social-system leaders were informed both by these insights and the best-available evidence. A final limitation to consider is that while we generated key insights from the deliberations using an iterative approach that is grounded in qualitative methods, we did not conduct a full qualitative analysis that included coding of transcripts. For the citizen panels, we used an iterative approach to identify key themes within and between panels and used recordings to corroborate our detailed notes. Detailed qualitative analysis was, however, not possible for the stakeholder dialogue given that it adopted the Chatham House Rule, which precludes recording of the deliberations.

## Conclusions

Action will require a national learning collaborative to support spread and scale of successful prevention, care and support initiatives. Moreover, this type of approach will help operationalize, in at least one sector, the recommendations from the GCESC that emphasizes the need for more coordinated evidence-support systems [24, 25], and doing so in a way that enables rapid-learning and improvement cycles to support spread and scale of promising new system-level innovations.

## Data Availability

This manuscript presents results from a series of citizen panels and a national stakeholder dialogue in Canada. All of the reports that this manuscript presents results from are publicly available here: https://www.mcmasterforum.org/find-evidence/products/project/enhancing-the-delivery-of-comprehensive-care-for-people-living-with-hiv-in-canada.

## Abbreviations

HIV: Human immunodeficiency virus
STBBI: Sexually transmitted and blood-borne infections
UNAIDS: Joint United Nations Programme on HIV/AIDS
GCESC: Global Commission on Evidence to Address Societal Challenges
